# High glucose protects cardiomyocytes against ischaemia/reperfusion injury by suppressing myocardiocyte apoptosis via circHIPK3/miR‐29b/AKT3 signalling

**DOI:** 10.1111/jcmm.16527

**Published:** 2021-05-05

**Authors:** Nan Cheng, Chang Jin, Ping Jin, Dan Zhu, Zuoxu Hou

**Affiliations:** ^1^ Department of Cardiovascular Surgery Chinese PLA General Hospital Beijing China; ^2^ Medical Innovation Research Division Research Center for Biomedical Engineering Chinese PLA General Hospital Beijing China; ^3^ Department of Cardiovascular Surgery The First Affiliated Hospital of Air Force Military Medical University Xi'an China; ^4^ Department of Arrhythmia Qinghai Cardio‐Cerebrovascular Hospital Xi'ning China; ^5^ Department of Thoracic Cardiovascular Surgery General Hospital of Central Theater Command of the People's Liberation Army Wuhan China

**Keywords:** AKT3, apoptosis, circHIPK3, high glucose, ischaemia/reperfusion, miR‐29b

## Abstract

High glucose promoted expression of AKT3, a direct target gene of miR‐29b, by regulating circHIPK3 that functioned as ceRNA to sponge and down‐regulate miR‐29b. As a potential target gene of miR‐29b, AKT3 plays a crucial role in the pathogenesis of myocardial ischaemia/reperfusion (I/R) injury, and this study aimed to investigate the potential role of high glucose in the outcome of I/R injury. qPCR and luciferase assay were carried out to investigate the relationship between the expression of circHIPK3, miR‐29b and ATK3 mRNA. Immunohistochemistry and TUNEL were performed to analyse the relationship between AKT3 expression and apoptosis of myocardiocytes in vivo. No obvious difference in myocardial functions was observed between I/R and control rats under hyperglycaemia (HG) and normal glucose (NG) conditions, except that the infarct size/area at risk (IS/AR) ratio and the amount of h‐FABP expression were different under HG and NG conditions. The expression of circHIPK3 and ATK3 was significantly elevated in the rats preconditioned by NG, whereas the expression of miR‐29a was remarkably decreased. Meanwhile, the apoptosis of myocardial tissue was reduced in the rats preconditioned by NG. Luciferase assay confirmed that miR‐29a played a repressive role in the expression of circHIPK3 and ATK3. And subsequent study indicated that the over‐expressed AKT3 could rescue the increased cell apoptosis rate induced by the knockdown of circHIPK3. In this study, we demonstrated that high glucose protects cardiomyocytes against I/R associated injury by suppressing apoptosis and high glucose promoted the expression of AKT3 by regulating the expression of circHIPK3/miR‐29b.

## 1 INTRODUCTION

As a type of injuries usually caused by the initial occlusion of blood vessels and subsequent damage to the blood vessels during blood flow restoration, which can often result in significant mechanical stress on blood vessel wall, I/R injury can lead to significant morbidity as well as mortality after surgical operations.^1^, ^2^ As a complex disorder, I/R injury can lead to heart function impairment as well as reduced benefit of reperfusion carried out to solve myocardial infarction.^2^ So far, multiple factors, such as local inflammation, dysfunction of the micro‐vasculature, disruption in the homeostasis of Ca2+, production of reactive oxygen species (ROS), as well as the activation of signalling pathways involved in the apoptosis, have been shown to contribute to the onset of I/R injury.^3^ In addition, high glucose level can reduce the tolerance to ischaemia while increasing the severity of I/R injury.^4^, ^5^ Furthermore, I/R injury will decrease the health of cells by increasing the level of oxidative stress, and the exposure of cells to an HG condition can further increase the level of oxidative stress.^6^

The level of circHIPK3 in HAECs and HUVECs can be reduced by culturing the cells under an HG condition. More importantly, the increased expression of circHIPK3 in HAECs and HUVECs apparently suppressed the growth and survival of endothelial cells while promoting the apoptosis of these cells. In contrary, the silencing of circHIPK3 expression by using specific siRNA sequences further promoted the apoptosis of endothelial cells already induced by the HG condition.^7^

By splicing the transcripts of RNAs, a new class of RNAs named circular RNAs (circRNAs) can be produced. As members of non‐coding RNAs (ncRNAs) endogenously present in many types of cells, circRNAs can compete with their target microRNA (miRNA) to alter the expression of miRNA.^8^, ^9^ Recently, it was shown that DM rats are associated with dysregulation of circRNAs.^10^, ^11^, ^12^ Another recent article showed abundant expression of circHIPK3 in both human tissues and tissues of other animal species.^13^ In particular, circHIPK3 can regulate the growth of cells as well as their apoptosis and migration by competing with the activity of several miRNAs in both normal cells as well as in tumour cells, such as the cells of colorectal carcinoma and hepatocellular cancer.^14^, ^15^, ^16^, ^17^

A recent study showed that circHIPK3 can play a regulatory role by sponging the function of miR‐29b‐3p to decrease the expression levels of several genes such as a‐SMA, COL3A1, as well as COL1A1.^18^ In addition, the elevated expression of miR‐29 gene was confirmed to play a certain therapeutic role in the treatment of multiple fibrotic disorders, including the fibrosis in the heart, lung and kidney, as well as liver.^19^, ^20^, ^21^ Furthermore, miR‐29 can also act as a promoter of myogenesis via inhibiting the feedback signal of a transcription factor termed YY1.^22^ Moreover, the enhancement of AKT signalling has been frequently discovered in cancer patients. For example, by using bioinformatic tools, it was shown that miR‐29b plays an essential role in regulating the expression levels of AKT2 as well as AKT3. Similarly, miR‐29b can also suppress the progress of glycolysis by inhibiting the signalling of both PKM2 and AKT‐HK2 in cells of ovarian cancer, suggesting a critical role of miR‐29b in controlling the metabolism of glucose in cancer cells.^23^

It has been shown that HG may alter the expression of circHIPK3, and circHIPK3 may regulate the expression of miR‐29b.^7^, ^24^ Furthermore, as a possible target gene of miR‐29b, AKT3 has been reported to be involved in the pathogenesis of myocardial I/R.^25^ In this study, we set up an animal model of I/R to study the effect of HG on the signalling pathway of circHIPK3/miR‐29b/AKT3/apoptosis in the pathogenesis of myocardial I/R.

## 2 MATERIALS AND METHODS

### 2.1 Animal grouping and treatment

In this study, 32 male adult Wistar rats (with an average bodyweight of 250‐300 g) were acquired from our experimental animal centre and then given purified water at ad libitum along with a regular diet. After 7 days of adaptation, the rats were randomly divided into four groups with eight rats in each group, that is (a) control normoglycaemic group (C‐NG group); (b) I/R injury preconditioned normoglycaemic group (PC‐NG group); (c) control hyperglycaemic group (C‐HG group); and (d) I/R injury preconditioned hyperglycaemic group (PC‐HG group).

To collect heart tissues, the rats were first placed under anaesthesia with ip injection 50 mg/kg of sodium pentobarbitone in conjunction with heparin. In the next step, the heart was rapidly removed and dipped into a perfusion buffer equilibrated at 4°C. Then, the heart was cannulated through the aorta and subsequently perfused at a 75 mm Hg perfusion pressure using a KH solution (pH 7.4, containing 118.0 mmol/L of NaCl, 3.2 mmol/L of KCl, 1.2 mmol/L of MgSO_4_, 25.0 mmol/L of NaHCO_3_, 1.18 mmol/L of KH_2_PO_4_, 2.5 mmol/L of CaCl_2_ and 11.1 mmol/L of glucose). In the C‐NG and C‐HG groups, the collected heart tissues were subjected to the occlusion of the left anterior descending (LAD) coronary artery for 30 minutes and subsequent reperfusion of 120 minutes carried out by releasing the clamp used to induce occlusion. In the PC‐HG and PC‐NG groups, the collected heart tissues were exposed to two cycles of ischemic PC, which was carried out by inducing the occlusion of LAD coronary artery for 5 minutes and subsequent reperfusion of another 5 minutes.

In this study, the pressure in the left ventricle of each heart was determined by utilizing a balloon filled with water and inserted into the left ventricle. The balloon was inflated to get a 7 mm Hg end‐diastolic pressure before it was linked to the pressure transducer. In addition, the +d*P*/d*t*_max_, index of contraction, maximal rate of pressure development, coronary flow, heart rate (HR), −d*P*/d*t*_max_, diastolic pressure in the left ventricle, systolic pressure in the left ventricle, LV developed pressure (LVDP) and index of relaxation were measured by utilizing the Power‐Lab/8SP Chart 7 software (ADInstruments).

All animal operations in this study were carried out in strict compliance with the Guide for the Care and Use of Laboratory Animals issued by the NIH and Institutional ethical committee has approved the protocol of this study.

### 2.2 Measurement of infarct size

In this study, the area at risk (AR) size and the infarct size (IS) were determined by utilizing double staining using 2,3,5‐triphenyltetrazolium chloride (TTC) as well as KMnO4 following the protocol described in a previous paper.^26^

### 2.3 Measurement of h‐FABP release from the heart

After the establishment of I/R injury, the effluent from the heart was collected and then randomly divided into six portions that were 20 minutes apart. Two millilitres of the sample taken from each portion of the effluent was dialyzed at 4°C against double distilled water overnight by utilizing a cellulose membrane connected to a dialysis piping. After dialysis, 1 mL of the dialyzed sample was taken, lyophilized into powders, and then dissolved in 100 µL of a sample diluent. In the next step, the solution was analysed by utilizing an ELISA assay kit of rat h‐FABP in accordance with the protocol provided by the kit manufacturer. The measured concentration value of h‐FABP in each sample was then multiplied by utilizing the volume of collected effluent to calculate the content of h‐FABP in the effluent obtained from each heart.

### 2.4 RNA isolation and real‐time PCR

The total RNA content in collected tissue and cell samples was isolated first by utilizing a TRIzol reagent (Invitrogen). In the next step, the reverse transcription of isolated RNA to cDNA was carried out by utilizing a cDNA synthesis kit (Exiqon) in accordance with the protocol provided by the kit manufacturer. Then, the real‐time PCR reaction was carried out by utilizing an ExiLENT SYBR Green real‐time PCR kit (Exiqon) in accordance with the protocol provided by the kit manufacturer. The real‐time PCR was carried out on a Bio‐Rad real‐time PCR instrument (Bio‐Rad laboratories). After the real‐time PCR was completed, the relative expression of circHIPK3, miR‐29b and AKT3 mRNA was calculated using the delta‐CT method, and the expression of housekeeping gene U6 and GAPDH was utilized to normalize the results.

### 2.5 Cell culture and transfection

H9C2 cells were maintained in a DMEM medium (Gibco, Thermo Fisher Scientific) added with 10% FBS and necessary antibiotics. Then, the cells were randomly divided into four groups, that is (a) Normoxic and normoglycaemic group (NX‐NG group); (b) Reoxidation and normoglycaemic group (HR‐NG group); (c) Normoxic and hyperglycaemic group (NX‐HG group); and (d) Reoxidation and hyperglycaemic group (HR‐HG group). At first, the cells were exposed to a hypoxic condition at 37°C induced by utilizing the D‐Hank solution containing 4.166 mol/L of NaHCO_3_, 5.37 mmol/L of KCl, 136.89 mmol/L of NaCl, 0.44 mmol/L of KH_2_PO_4_, 5 mmol/L of d‐glucose and 0.338 mmol/L of Na_2_HPO_4_ (pH 7.4). After the 48 hours induction of hypoxia was completed, the culture media were quickly replaced by utilizing a fresh DMEM. In the normoglycaemic groups, the cells were exposed to a 5.0 mmol/L concentration of glucose for 48 hours. In the hyperglycaemic groups, the cells were exposed to a 15 mmol/L concentration of glucose for 48 hours. For the transfection experiment, the cells were cultured to 70% confluence and then transfected with corresponding vectors using Lipofectamine 3000 (Invitrogen) in accordance with the protocol provided by the manufacturer.

Also, H9C2 cells were divided into four groups to evaluate the effect of circHIPK3 on the apoptosis in vitro, that is (a) Cells transfected with NC siRNA group (NC siRNA group); (b) Cells transfected with circHIPK3 siRNA group (circHIPK3 siRNA group); (c) Cells co‐transfected with circHIPK3 siRNA and empty pcDNA vector group (circHIPK3 siRNA +pcDNA group); and (d) Cells transfected with circHIPK3 siRNA and pcDNA overexpressing AKT3 (circHIPK3 siRNA+pcDNA‐AKT3 group).

### 2.6 Vector construction, mutagenesis and luciferase assay

Our results of sequence analysis showed that both the 3′ UTR of circHIPK3 and AKT3 contained potential binding sites of miR‐29b. In order to further explore the regulatory relationship between circHIPK3 and miR‐29b, as well as between miR‐29b and AKT3, we established luciferase vectors containing wild‐type and mutant 3′ UTR of circHIPK3 and AKT3 as follows: First, the wild‐type fragments of 3′ UTR of circHIPK3 and AKT3 containing the binding sites of miR‐29b were cloned into separate pcDNA3.1 luciferase reporter gene vectors (Promega) downstream of the luciferase gene to generate wild‐type vectors of circHIPK3 and AKT3, respectively. In the next step, the miR‐29b binding sites in the 3′ UTR of wild‐type circHIPK3 and AKT3 were subjected to site‐directed mutagenesis, respectively, carried out by using a Quick Change II mutagenesis kit (Stratagene) in accordance with the protocol provided by the kit manufacturer. Then, the mutant type fragments of 3′ UTR of circHIPK3 and AKT3 containing the mutated binding sites of miR‐29b were also cloned into different pcDNA3.1 luciferase reporter gene vectors downstream of the luciferase gene to generate mutant vectors of circHIPK3 and AKT3, respectively. Thereafter, H9C2 cells were cultured to 70% confluence and then co‐transfected with mutant or wild‐type vectors of circHIPK3 and AKT3 in conjunction with miR‐29b using Lipofectamine 3000 in accordance with the protocol provided by the manufacturer. At 48 hours post‐transfection, the luciferase reporter assays were carried out using a Dual Luciferase Assay kit (Promega) in accordance with the protocol provided by the kit manufacturer to determine the luciferase activity of transfected cells.

### 2.7 Immunohistochemistry

Collected tissue samples were fixed by utilizing 4% formaldehyde, rinsed by PBS, permeabilized at room temperature by utilizing 0.1% Triton X‐100, blocked for 15 minutes at room temperature by utilizing 3% BSA, and then incubated with anti‐AKT3 primary antibody (Millipore) and fluorescein isothiocyanate‐tagged secondary antibodies (Thermo Fisher Scientific) in accordance with the protocol provided by the manufacturer. After counter staining at room temperature for 5 minutes with 4′,6‐diamidino‐2‐phenylindoledihydrochloride (DAPI), the relative expression of AKT3 protein in each sample was analysed under an IX71 fluorescence microscope (Olympus).

### 2.8 TUENL assay

The collected myocardial tissue samples were subjected to TUNEL assay to observe the apoptotic status. The samples were stained using a TUNEL kit (Beyotime Biotechnology) following the protocol provided by the kit manufacturer, and the stained samples were processed following common routine. Finally, the processed samples were observed under a fluorescence microscope, with 10 visual fields being randomly selected to calculate the ratio of positive cells and the apoptotic index.

### 2.9 Apoptosis analysis

H9C2 cells were processed and fixed in 70% prechilled ethanol following common routine. After rinsed with PBS, the cells were incubated with 10 μL Annexin V‐FITC and 5 μL PI in the dark at room temperature for 15 minutes. Flow cytometry was performed to measure cell apoptosis at 488 mm.

### 2.10 Statistical analysis

All experimental results were shown by means ± SE The statistical significance of inter‐group differences was evaluated using one‐way ANOVA. All statistical analyses were carried out using SPSS 21.0 (IBM). The level of statistical significance was set to *P* < .05.

## 3 RESULTS

### 3.1 Characterization of myocardial functions of isolated hearts under different conditions

**FIGURE 1:**
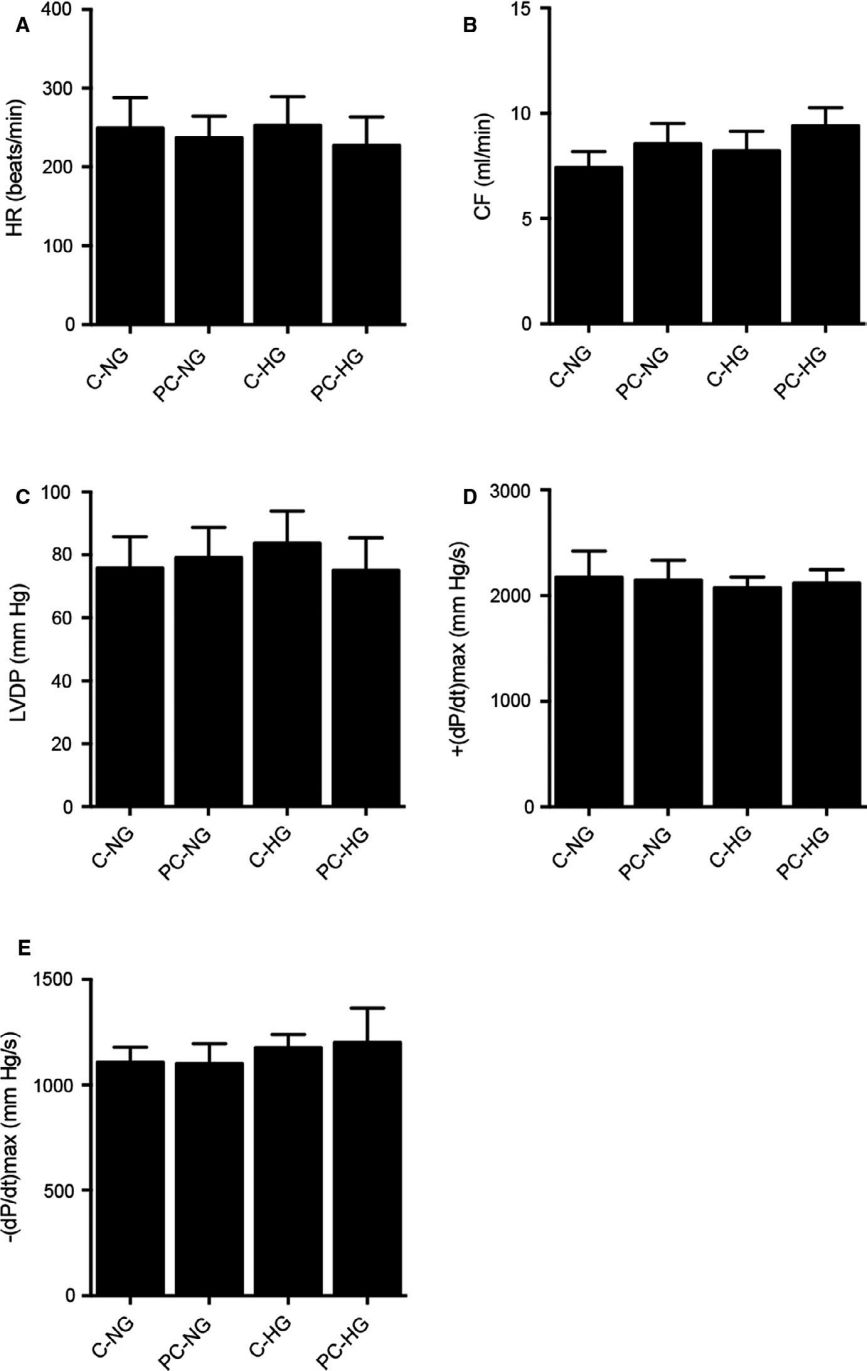
Values of myocardial parameters in the preconditioned and non‐preconditioned rats treated under normal glucose (NG) and hyperglycaemia (HG) conditions. A, Heart rates in the preconditioned and non‐preconditioned rats treated under NG and HG conditions. B, Coronary flow in the preconditioned and non‐preconditioned rats treated under NG and HG conditions. C, Left ventricular developed pressure in the preconditioned and non‐preconditioned rats treated under NG and HG conditions. D, Maximal rate of pressure development (+(d*P*/d*t*)_max_) in the preconditioned and non‐preconditioned rats treated under NG and HG conditions. E, Maximal rate of pressure fall (−(d*P*/d*t*)_max_) in the preconditioned and non‐preconditioned rats treated under NG and HG conditions

Control and ischaemia/reperfusion (IR)‐conditioned rats were subjected to hyperglycaemia (HG) and normal glucose (NG) treatments, respectively, before a series of myocardial parameters were measured. No obvious differences were observed in terms of the heart rate (HR, Figure 1A), coronary flow (CF, Figure 1B), left ventricular developed pressure (LVDP, Figure 1C), maximal rate of pressure development (+(d*P*/d*t*)_max_, Figure 1D) and maximal rate of pressure fall (−(d*P*/d*t*)_max_, Figure 1E) between the two groups.

### 3.2 Different infarct size/area at risk ratio and h‐FABP release in the preconditioned and non‐preconditioned rats treated under NG and HG conditions

**FIGURE 2:**
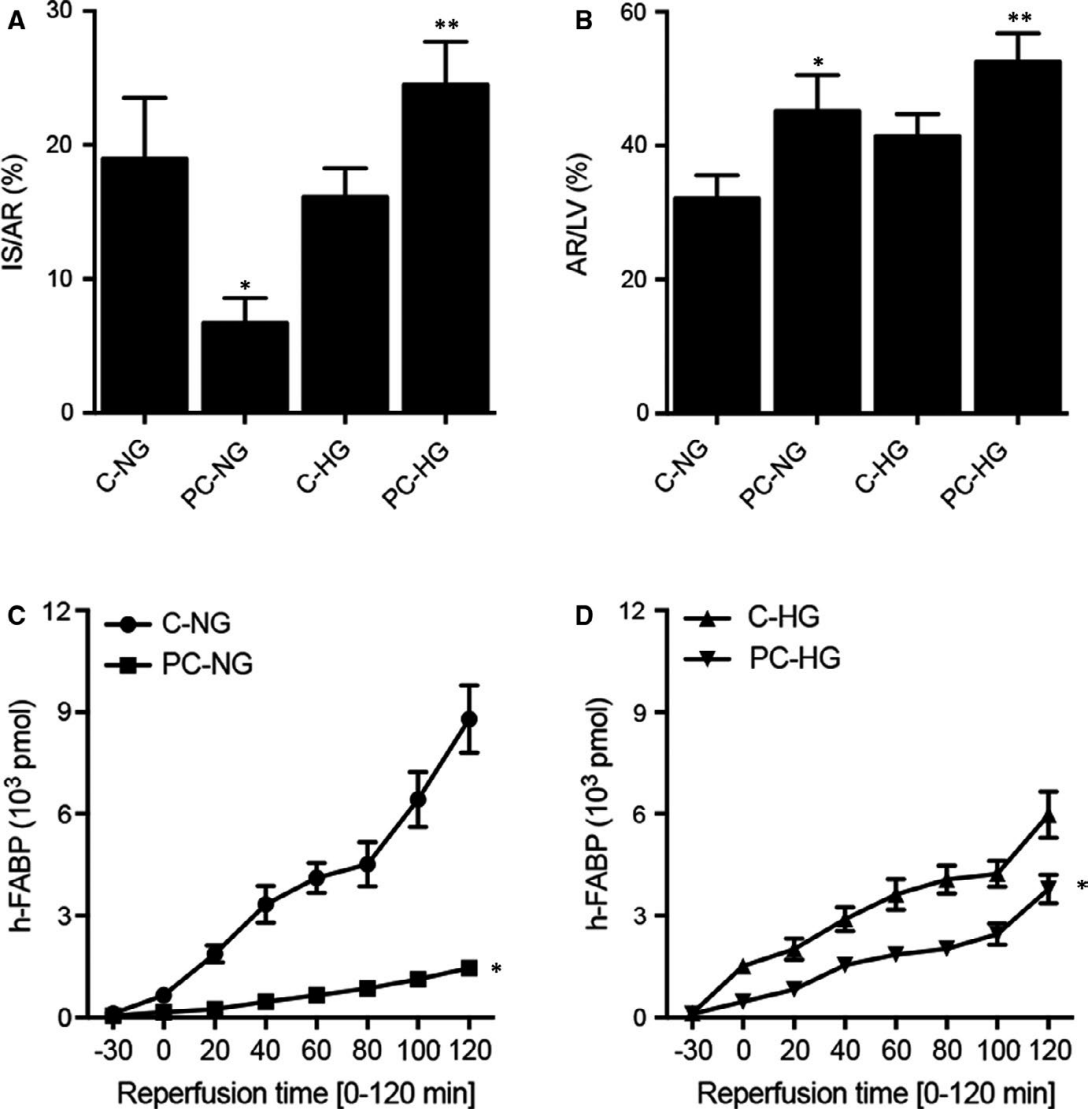
Differential infarct size/area at risk ratio and h‐FABP release in the preconditioned and non‐preconditioned rats treated under normal glucose (NG) and hyperglycaemia (HG) conditions (*P value < .05 vs C‐NG; **P value < .05 vs C‐HG). A, Infarct size/area at risk (IS/AR) ratio in the preconditioned and non‐preconditioned rats treated under NG and HG conditions. B, Area at risk/left ventricle size (AR/LV) ratio in the preconditioned and non‐preconditioned rats treated under NG and HG conditions. C, Cumulative h‐FABP release from the heart was notably increased in the non‐preconditioned rats. D, Cumulative h‐FABP release from the heart was notably increased in the preconditioned rats

Under the normal glucose condition, the infarct size/area at risk (IS/AR) ratio was significantly decreased in the I/R preconditioned rats. However, under the HG condition, the IS/AR ratio was remarkably increased in the I/R preconditioned rats. For the control rats, there was no obvious difference in the IS/AR ratio between NG and HG conditions. However, for the preconditioned rats, the IS/AR ratio was dramatically elevated (Figure 2A). For the area at risk/left ventricle size (AR/LV) ratio, no obvious difference was observed among different groups (Figure 2B). Under the NG condition, the amount of h‐FABP released from the heart was notably increased in the non‐preconditioned rats (Figure 2C). On the contrary, under HG condition, the amount of h‐FABP released from the heart was remarkably higher in the preconditioned rats (Figure 2D).

### 3.3 Differential expression of circHIPK3, miR‐29b and AKT3 mRNA in the preconditioned and non‐preconditioned rats treated under NG and HG conditions

**FIGURE 3:**
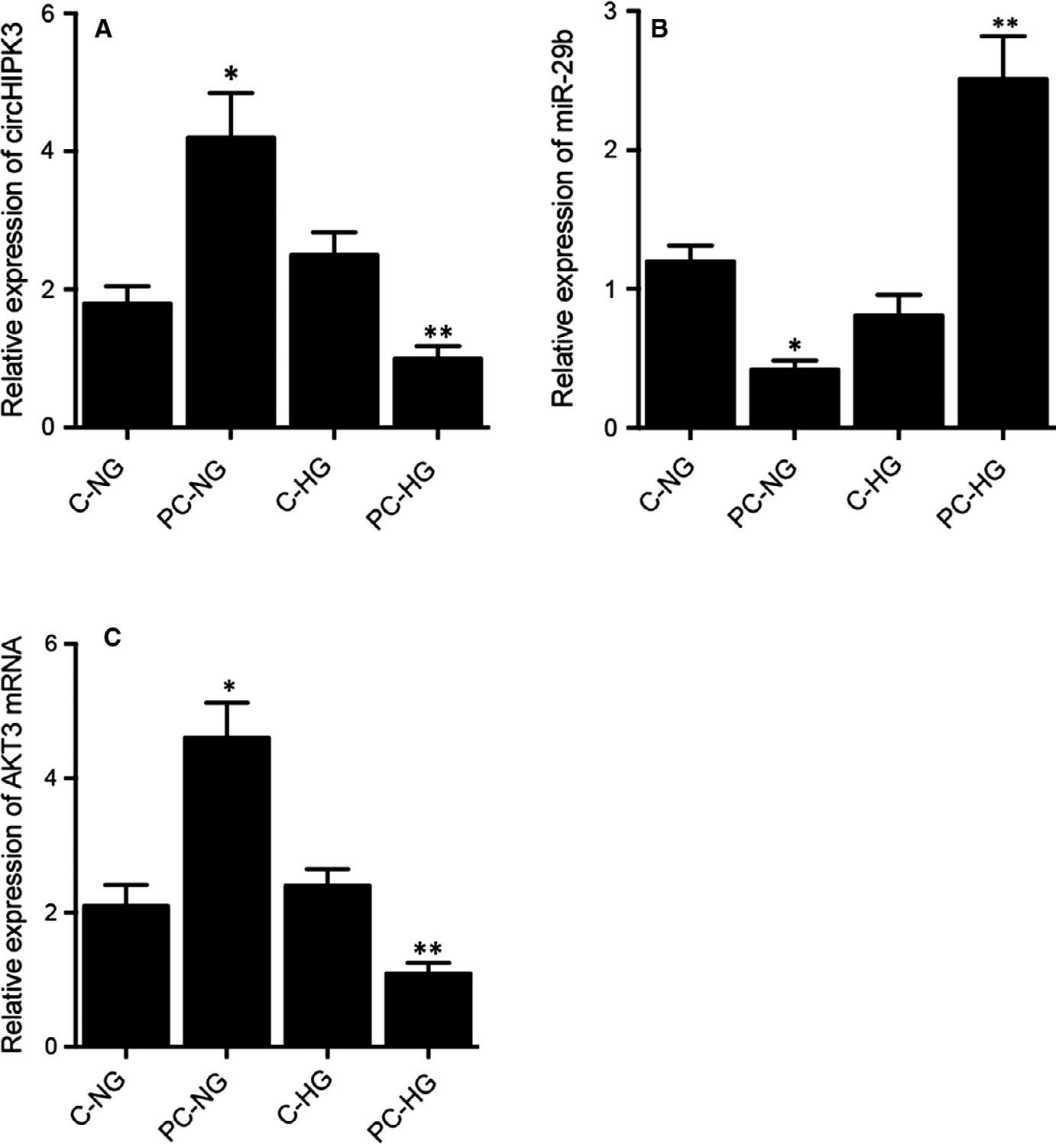
Differential expression of circHIPK3, miR‐29b and AKT3 mRNA in the preconditioned and non‐preconditioned rats treated under NG and HG conditions (*P value < .05 vs C‐NG; **P value < .05 vs C‐HG). A, Differential expression of circHIPK3 in the preconditioned and non‐preconditioned rats treated under NG and HG conditions. B, Differential expression of miR‐29b in the preconditioned and non‐preconditioned rats treated under NG and HG conditions. C, Differential expression of AKT3 mRNA in the preconditioned and non‐preconditioned rats treated under NG and HG conditions

**FIGURE 4:**
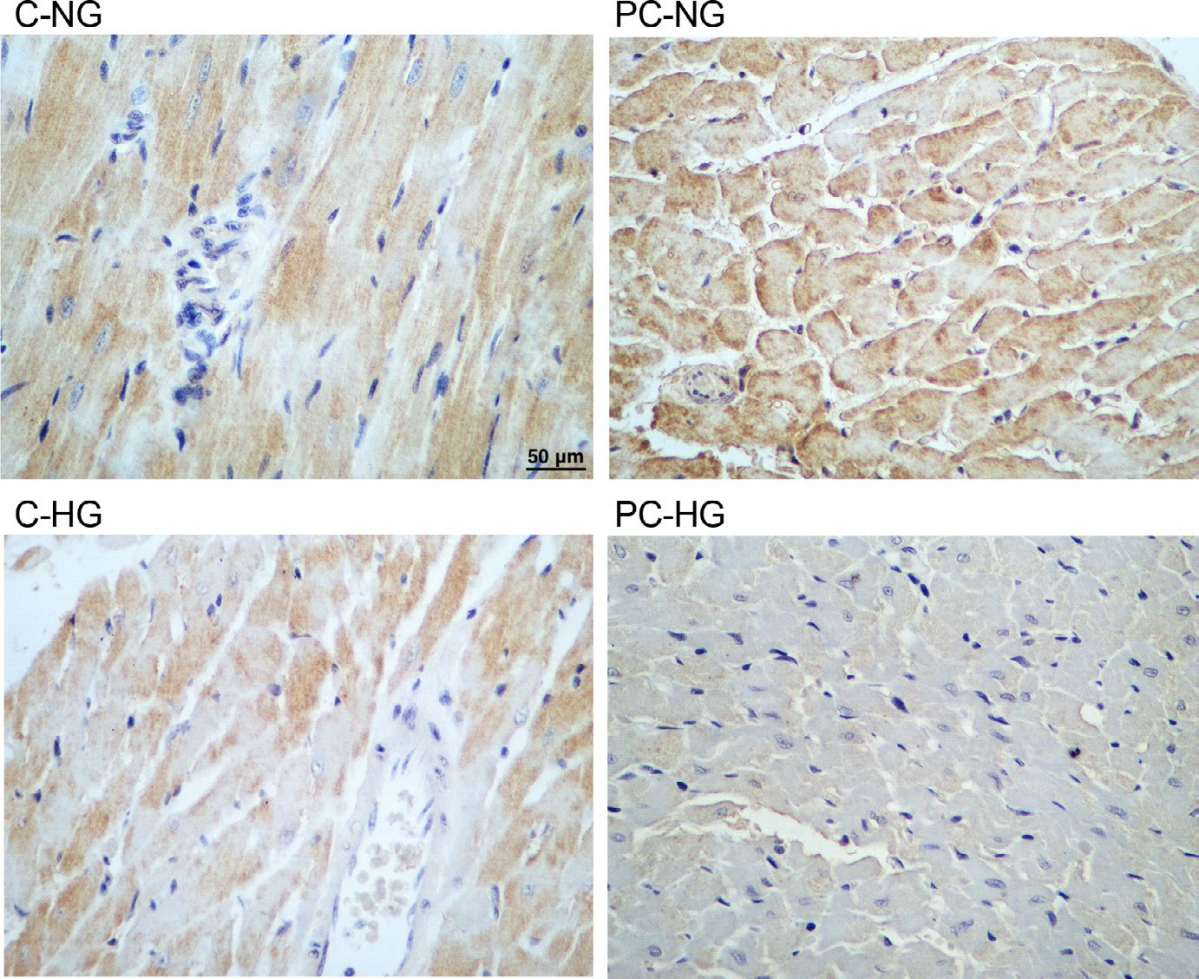
Immunohistochemistry analysis showed the differential AKT3 protein expression in the preconditioned and non‐preconditioned rats treated under NG and HG conditions (magnification × 200; scale bar = 50 μm)

Real‐time PCR was carried out to analyse the expression of circHIPK3, miR‐29b and AKT3 mRNA under different conditions. The expression of circHIPK3 was the highest in PC rats treated under NG condition and decreased gradually in non‐PC control rats treated under HG condition, followed by control rats treated under NG condition, while the preconditioned rats treated under HG condition showed the lowest expression of circHIPK3 (Figure 3A). In contrary to the trend of circHIPK3 expression, the expression of miR‐29b was the lowest in the PC rats treated under NG condition and the highest in the preconditioned rats treated under HG condition (Figure 3B). Moreover, the expression of AKT3 mRNA showed the same trend as that of circHIPK3 (Figure 3C). We further performed immunohistochemistry to evaluate the expression of AKT3 protein in the myocardial tissues, and the expression of AKT3 protein showed the same trend as that of AKT3 mRNA (Figure 4).

### 3.4 Different levels of apoptosis of myocardial tissues in preconditioned and non‐preconditioned rats treated under NG and HG conditions

**FIGURE 5:**
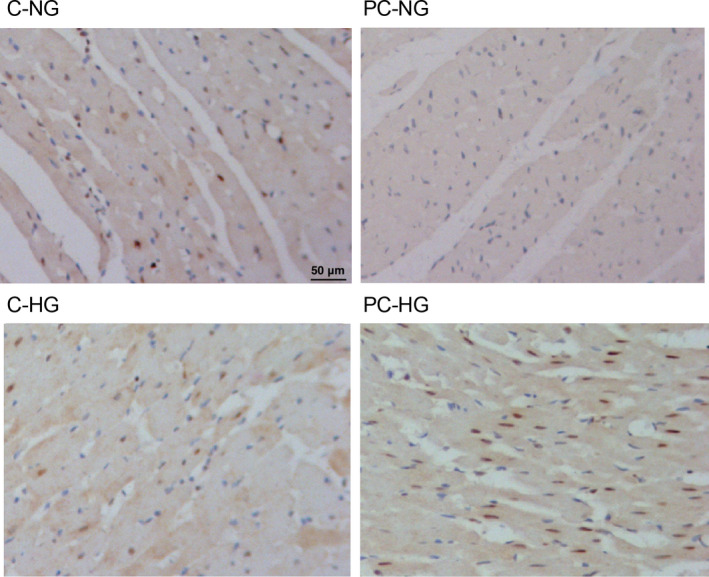
TUNEL assay demonstrated the different apoptotic status of myocardial tissues in the preconditioned and non‐preconditioned rats treated under NG and HG conditions (magnification × 400; scale bar = 50 μm)

TUNEL was carried out to evaluate the apoptotic status of myocardial tissues of rats treated under different conditions. The apoptotic level of myocardial tissues was the lowest in PC rats treated under NG condition and the highest in preconditioned rats treated under HG condition (Figure 5).

### 3.5 MiR‐29b repressed the luciferase activities of wild‐type circHIPK3 and ATK3 vectors by binding to their 3′ UTR

**FIGURE 6:**
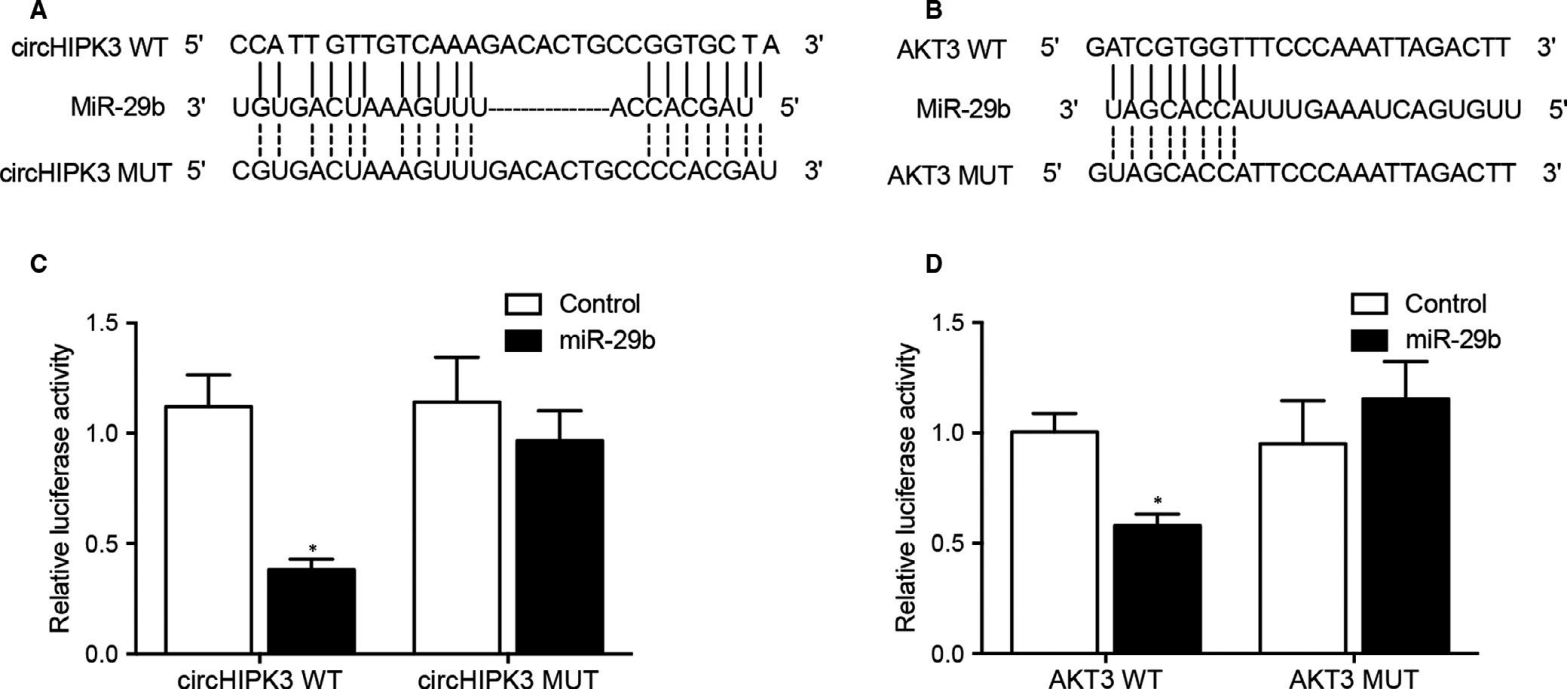
MiR‐29b inhibited the luciferase activity of circHIPK3 and ATK3 (*P value < .05 vs Control). A, Sequence analysis showed potential binding of miR‐29b to circHIPK3. B, The luciferase activity of wild‐type circHIPK3 was inhibited by miR‐29b. C, Sequence analysis showed potential binding of miR‐29b to ATK3. D, The luciferase activity of wild‐type ATK3 was repressed by miR‐29b

Sequence analysis showed that circHIPK3 (Figure 6A) and AKT3 (Figure 6C) contained potential binding sites for miR‐29b. In order to further explore the regulatory relationship between circHIPK3 and miR‐29b, and between miR‐29b and AKT3, we established luciferase vectors containing wild‐type and mutant circHIPK3 and AKT3, which were then co‐transected into cells with miR‐29b to check the regulatory effect of miR‐29b on the luciferase activity of circHIPK3 and AKT3. The luciferase activities of wild‐type circHIPK3 (Figure 6B) and AKT3 (Figure 6D) were significantly inhibited by miR‐29b, but the luciferase activities of mutant circHIPK3 and AKT3 were unaffected by miR‐29b.

### 3.6 Differential expression of circHIPK3, miR‐29b and AKT3 mRNA in H9C2 cells treated under different conditions

**FIGURE 7:**
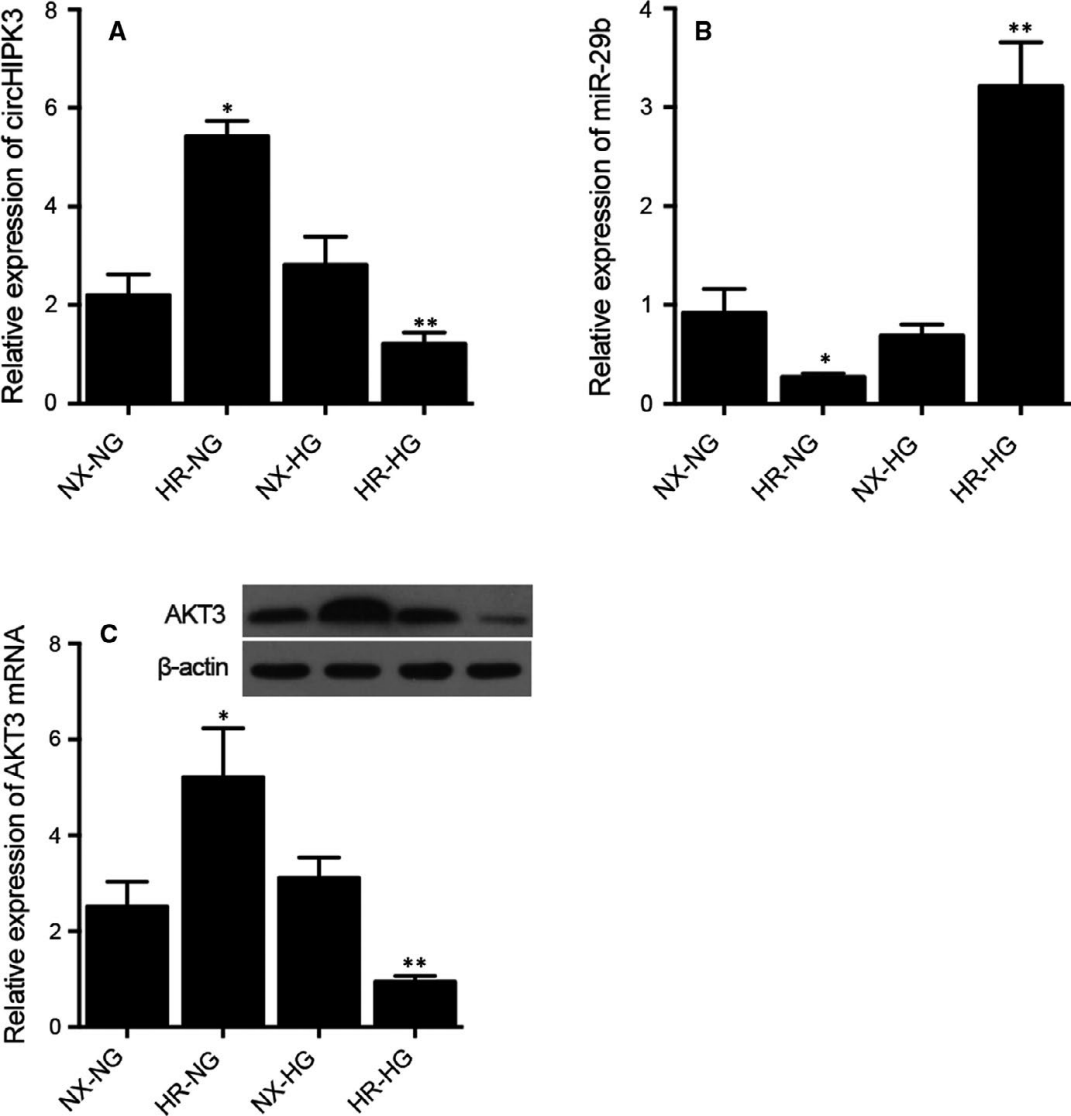
Differential expression of circHIPK3, miR‐29b and AKT3 mRNA in the H9C2 cells treated under NG and HG conditions (*P value < .05 vs NX‐NG; **P value < .05 vs NX‐HG). A, Differential expression of circHIPK3 in the H9C2 cells treated under NG and HG conditions. B, Differential expression of miR‐29b in the H9C2 cells treated under NG and HG conditions. C, Differential expression of AKT3 mRNA in the H9C2 cells treated under NG and HG conditions

H9C2 cells were cultured under different conditions before qPCR was performed to analyse the expression of circHIPK3, miR‐29b and ATK3. The expression of circHIPK3 (Figure 7A) and ATK3 (Figure 7C) was the highest in H9C2 cells treated under HR‐NG condition and the lowest in the H9C2 cells treated under HR‐HG condition. As expected, the expression of miR‐29b showed an opposite trend (Figure 7B).

### 3.7 Effect of circHIPK3 on cell apoptosis and ectopic expression of AKT3 in H9C2 cells treated under different conditions

**FIGURE 8:**
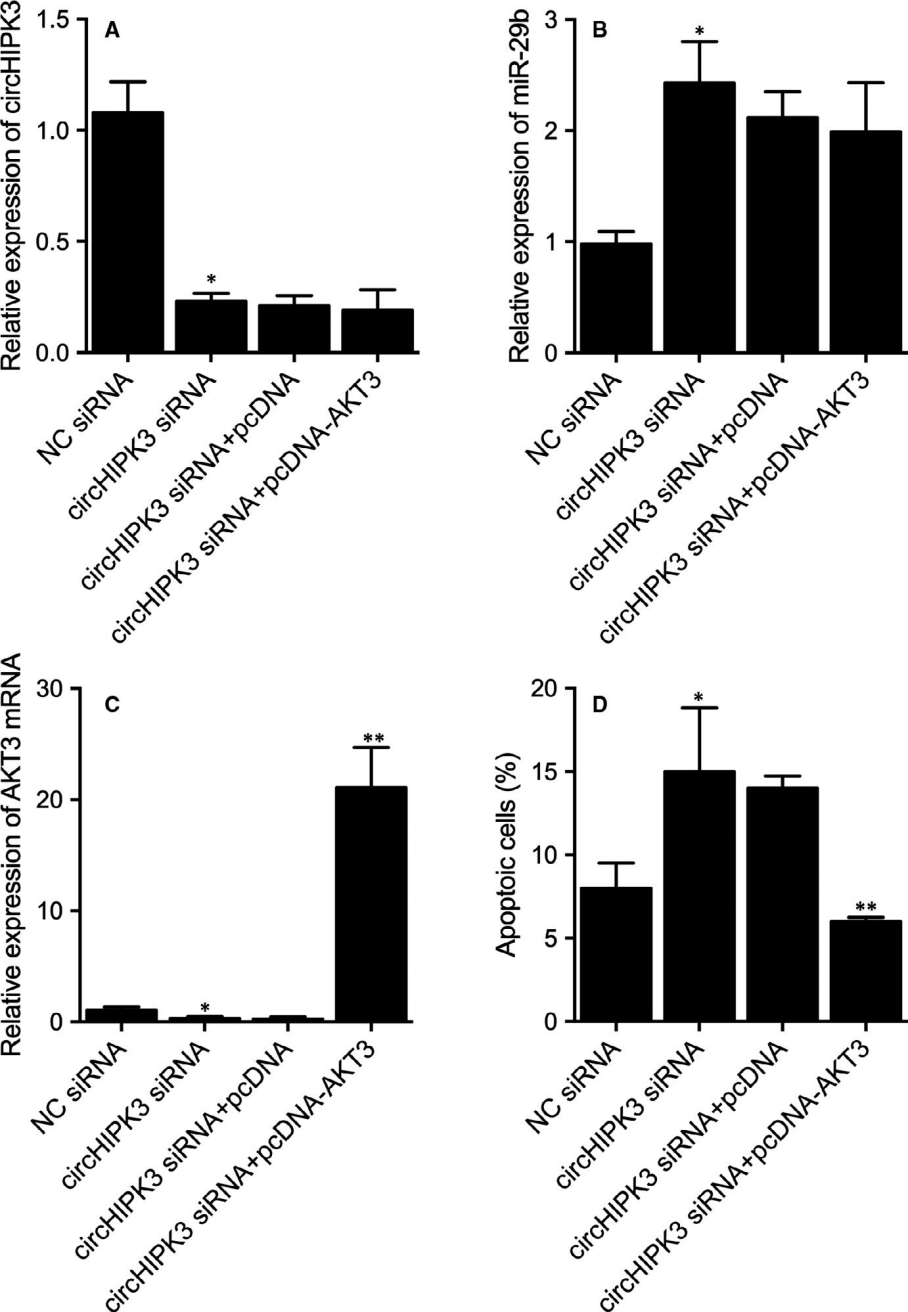
Differential expression of circHIPK3, miR‐29b and AKT3 mRNA and cell apoptosis rate in the H9C2 cells transfected with NC siRNA, circHIPK3 siRNA, circHIPK3 siRNA+pcDNA, circHIPK3 siRNA+pcDNA_AKT3 (*P value < .05 vs NC siRNA; **P value < .05 vs circHIPK3 siRNA+pcDNA) A, Differential expression of circHIPK3 in the H9C2 cells transfected with NC siRNA, circHIPK3 siRNA, circHIPK3 siRNA+pcDNA, circHIPK3 siRNA+pcDNA_AKT3. B, Differential expression of miR‐29b in the H9C2 cells transfected with NC siRNA, circHIPK3 siRNA, circHIPK3 siRNA+pcDNA, circHIPK3 siRNA+pcDNA_AKT3. C, Differential expression of AKT3 mRNA in the H9C2 cells transfected with NC siRNA, circHIPK3 siRNA, circHIPK3 siRNA+pcDNA, circHIPK3 siRNA+pcDNA_AKT3. D, Differential apoptosis rate of the H9C2 cells transfected with NC siRNA, circHIPK3 siRNA, circHIPK3 siRNA+pcDNA, circHIPK3 siRNA+pcDNA_AKT3

H9C2 cells were, respectively, transfected NC siRNA, circHIPK3 siRNA, circHIPK3 siRNA+pcDNA and circHIPK3 siRNA+pcDNA_AKT3. The expression of circHIPK3, miR‐29b and AKT3 mRNA was then evaluated and compared. As shown by the results, the expression of circHIPK3 (Figure 8A) was evidently down‐regulated upon the transfected of circHIPK3 siRNA. And the expression of miR‐29b (Figure 8B) was up‐regulated upon the knockdown of circHIPK3 in H9C2 cells. Moreover, expression of AKT3 mRNA (Figure 8C) was evidently decreased in cells transfected with circHIPK3 siRNA, while the co‐existence of pcDNA_AKT3 greatly enhanced the expression of AKT3 mRNA. Similarly, the number of apoptotic cells (Figure 8D) increased upon the knockdown of circHIPK3, while the over‐expression of AKT3 rescued the increased cell apoptosis rate.

## 4 DISCUSSION

In this study, we established an I/R rat model and treated them under HG and NG conditions. We measured the release of IS/AR, AR/LV and h‐FABP from the heart of these rats. Different patterns of IS/AR, AR/LV and h‐FABP release were observed, revealing that hyperglycaemia up‐regulated the expression of circHIPK3 and ATK3 in these rats while down‐regulating the expression of miR‐29a.

As an important circRNA, circHIPK3 expression is reduced under HG conditions due to the increased level of miR‐124, thus down‐regulating the expression levels of several pro‐survival factors such as STAT3 as well as SphK1. On the other hand, the suppression of miR‐124 expression can protect endothelial cells against HG‐induced apoptosis, suggesting that the increase of miR‐124 expression following the down‐regulation of circHIPK3 expression acts as a critical mediator in the injury of endothelial cells induced by HG.^7^ Another article also confirmed that the down‐regulation of circHIPK3 expression can lead to apparent alleviation of cardiac fibrosis induced by angiotensin II. Since TGF‐β acts as an essential mediator downstream of the angiotensin II pathway, the expression level of circHIPK3 can be apparently elevated upon exposure to TGF‐β.^27^, ^28^ On the other hand, the activity of TGF‐β is reduced upon silencing of circHIPK3 expression, suggesting a key role of TGF‐β and circHIPK3 in the onset of hypertrophy as well as cardiac fibrosis.^29^, ^30^ In addition, the expression level of miR‐29 in GC is closely related to the expression level of circHIPK3, while the silencing of circHIPK3 expression in GC cells inhibited their growth.^31^ In this study, we evaluated the expression of circHIPK3, miR‐29a and ATK3 in H9C2 cells cultured under H/R conditions. Expression of circHIPK3 and ATK3 shared the same trend under different conditions, while the expression of miR‐29a showed an opposite trend.

In MI, myofibroblasts are derived from myocardial fibroblasts and such differentiation of myocardial fibroblasts can cause excessive accumulation of extracellular matrix.^32^ Melchiorbecker and his colleagues have shown that the expression level of miR‐29 is high in both myocardial fibroblasts and tissues suffering from myocardial fibrosis.^32^ In addition, van and his colleagues showed that the level of miR‐29 expression is reduced in rats suffering from MI.^33^ Moreover, Zhang and his colleagues showed that the increased level of miR‐29 expression can suppress the progression of cardiac fibrosis by targeting the TGF‐β/Smad3 pathway.^34^

The stimulation induced by a high level of glucose can modulate the progress of cell apoptosis, while the suppression of MIAT expression can reverse HG‐induced apoptosis. In addition, the knockdown of miR‐29b expression can apparently reverse the effect of MIAT in inducing cell apoptosis.^35^ Interestingly, hypoxia can also induce the injury of cardiomyocytes by promoting their apoptosis.^36^ In this study, we carried out luciferase assays to explore the regulatory role of miR‐29a in the expression of circHIPK3 and ATK3. The results demonstrated that miR‐29a showed an inhibitory effect on the expression of circHIPK3 and ATK3 by binding to their 3′ UTR.

As a signal downstream to PI3K, Akt plays an anti‐apoptotic role in PIK3R3/Akt3‐mediated I/R injury.^37^ In fact, both PIK3R3 and Akt3 play critical roles in the apoptosis as well as proliferation of cardiomyocytes.^25^, ^37^ More interestingly, miR‐181b‐5p can negatively regulate the expression of both PIK3R3 and Akt3 during I/R injury. Finally, the elevated levels of Akt3 and PIK3R3 expression can enhance cell growth while suppressing cell apoptosis, but the presence of miR‐181b‐5p reverses the effect of PIK3R3 and Akt3. Thus, the Akt3/PIK3R3 pathway acts as a key mediator in I/R injury.^25^

## 5 CONCLUSION

In conclusion, the findings of this study demonstrated that high glucose protects cardiomyocytes against I/R associated injury by suppressing apoptosis and high glucose promoted the expression of AKT3 by regulating the expression of circHIPK3/miR‐29b.

## CONFLICT OF INTEREST

The authors declare that they have no conflicts of interest.

## AUTHOR CONTRIBUTIONS

**Nan Cheng:** Conceptualization (equal); Formal analysis (equal); Investigation (equal); Writing–original draft (equal); Writing–review and editing (equal). **Chang Jin:** Investigation (equal); Methodology (equal); Resources (equal). **Ping Jin:** Formal analysis (equal); Investigation (equal); Visualization (equal). **Dan Zhu:** Investigation (equal); Resources (equal); Validation (equal). **Zuoxu Hou:** Conceptualization (equal); Funding acquisition (equal); Investigation (equal); Supervision (equal); Validation (equal); Writing–original draft (equal); Writing‐review and editing (equal).

## Data Availability

The data that support the findings of this study are available from the corresponding author upon reasonable request.
